# Supplementary material for: New PEPTIR-2.0 Peptide Designed for Use as Recognition Element in Electrochemical Biosensors with Improved Specificity towards E. coli O157:H7
Source: Molecules. 2022 Apr 22;27(9):2704. doi: 10.3390/molecules27092704 (PMC9105347; doi:10.3390/molecules27092704)
Supplement: Supplementary file 1 [file molecules-27-02704-s001.zip › molecules-1685116-supplementary.pdf]

Supplementary information

New PEPTIR-2.0 peptide designed for use as recognition element in electrochemical biosensors with improved specificity towards *E. coli* O157:H7

Jose Luis Ropero-Vega [1,*], Joshua Felipe Redondo-Ortega [1], Juliana Paola Rodríguez-Caicedo [1], Paola Rondón-Villarreal [2] and Johanna Marcela Flórez-Castillo [1]

1 Universidad de Santander, Facultad de Ciencias Naturales, Ciencias Básicas y Aplicadas Para la Sostenibilidad—CIBAS, Calle 70 No. 55-210, Santander, Bucaramanga C.P. 680003, Colombia; hugofelipez@hotmail.com (J.F.R.-O.); julianapao6@gmail.com (J.P.R.-C.); johanna.florez@udes.edu.co (J.M.F.-C.)

2 Universidad de Santander, Facultad de Ciencias Médicas y de la Salud, Instituto de Investigación Masira, Calle 70 No. 55-210, Santander, Bucaramanga C.P. 680003, Colombia; diseno.molecular@udes.edu.co

* Correspondence: jose.ropero@udes.edu.co or jlroperovega@gmail.com; Tel.: +57-7-6516500 (ext. 1665)

Table S1. Peptide sequences obtained from the modification of the original sequence of PEPTIR-1.0 (QKVNIDELGNAIPSGVLKDD).

Amino acid change	Sequence	RMSD
PEPTIR-1.0	QKVNIDELGNAIPSGVLKDD	**4.834**
Leucine	QKVNID**L**LGNAIPSGVLKDD	6.781
	QKVNI**L**ELGNAIPSGVLKDD	8.231
	QKVNIDELGNAIPSGVL**L**DD	7.548
	QKVNI**LL**LGNAIPSGVL**L**DD	**5.482**
Valine	QKVNID**V**LGNAIPSGVLKDD	5.49
	QKVNI**V**ELGNAIPSGVLKDD	8.074
	QKVNIDELGNAIPSGVL**V**DD	7.503
	QKVNI**VV**LGNAIPSGVL**V**DD	10.089
Glycine	QKVNID**G**LGNAIPSGVLKDD	5.723
	QKVNI**G**ELGNAIPSGVLKDD	6.64
	QKVNIDELGNAIPSGVL**G**DD	3.022
	QKVNI**GG**LGNAIPSGVL**G**DD	5.36
Alanine	QKVNID**A**LGNAIPSGVLKDD	7.518
	QKVNI**A**ELGNAIPSGVLKDD	**3.502**
	QKVNIDELGNAIPSGVL**A**DD	8.969
	QKVNI**AA**LGNAIPSGVL**A**DD	7.732
Methionine	QKVNID**M**LGNAIPSGVLKDD	8.097
	QKVNI**M**ELGNAIPSGVLKDD	7.537
	QKVNIDELGNAIPSGVL**M**DD	8.773
	QKVNI**MM**LGNAIPSGVL**M**DD	**4.861**

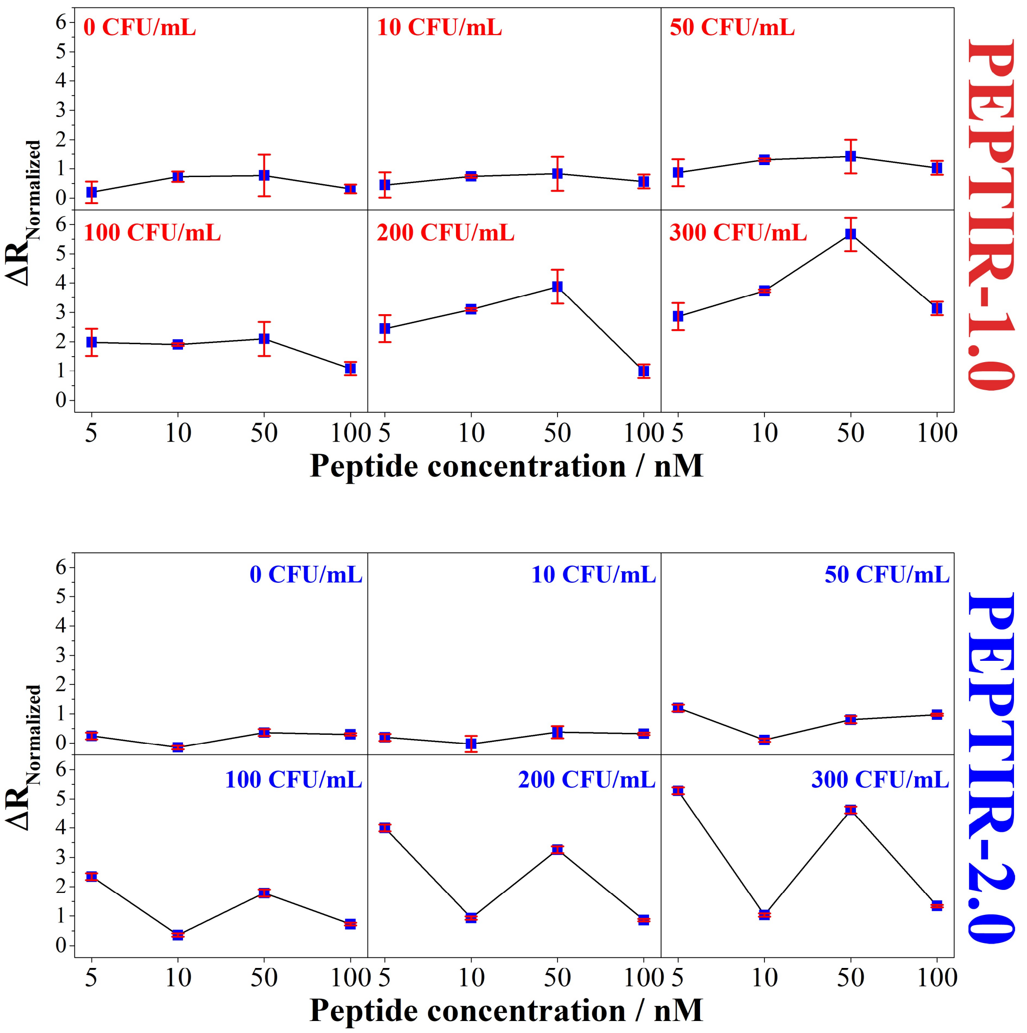

Figure S1. Electrochemical response of the biosensors represented as $\Delta R_{Normalized}$ as a function of the concentration of peptide in the solution of immobilization.

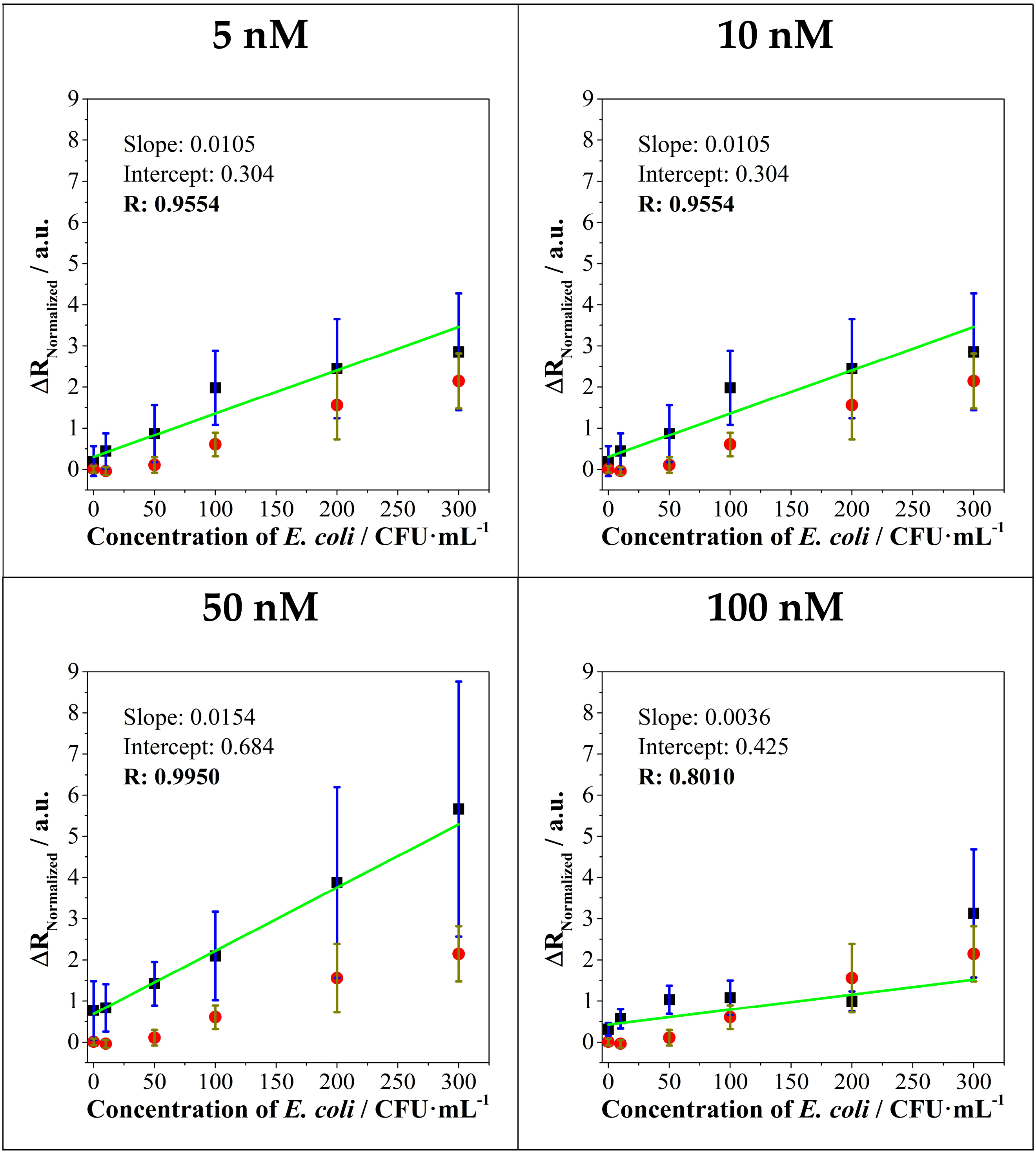

Figure S2. Calibration curves for PEPTIR-1.0.

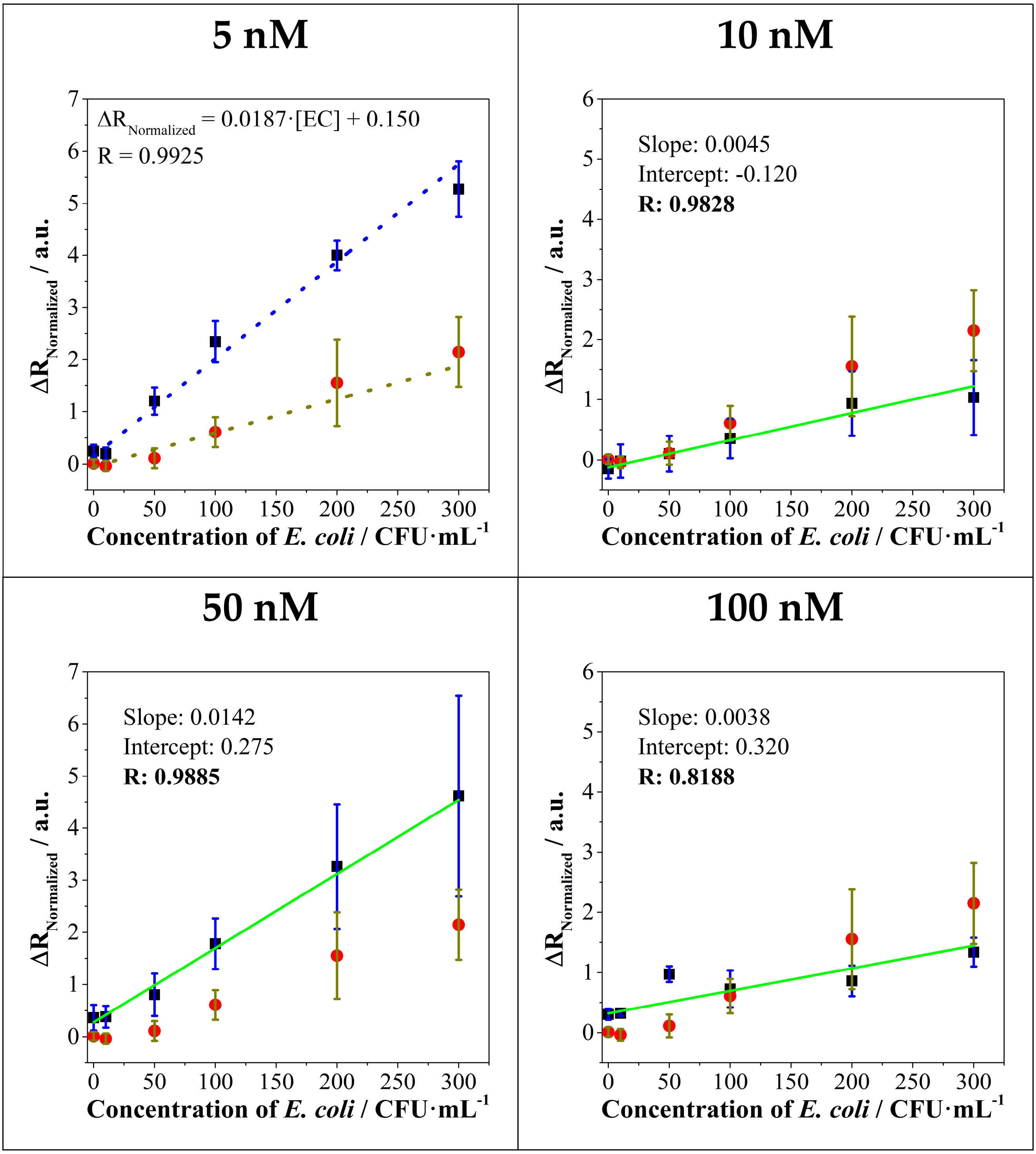

Figure S3. Calibration curves for PEPTIR-2.0.
